# Is Circumcision “Necessary” in Islam? A Philosophical Argument Based on Peer Disagreement

**DOI:** 10.1007/s10943-022-01635-0

**Published:** 2022-08-24

**Authors:** Hossein Dabbagh

**Affiliations:** Department of Continuing Education, University of Oxford, Oxford, UK

**Keywords:** Circumcision, Islam, Ethics, Peer disagreement, Harm

## Abstract

In recent years, there has been a resurgence in debates on the ethics of child genital cutting practices, both female and male, including within a Muslim context. Opponents of female genital cutting sometimes assert that the practice is not mentioned explicitly in the Qur’an as a way of implying that it does not have any religious standing within Islam. However, neither is male genital cutting mentioned explicitly in the Qur’an, and yet most people accept that it is a Muslim religious practice. Both practices, however, are mentioned in secondary sources of Islamic jurisprudence, with disagreement among religious authorities about the status or authenticity of some of these sources. This paper considers the religious status of both female and male genital cutting practices within Islam and employs a philosophical argument based on “peer disagreement” to ask whether either practice is necessary (i.e., religiously required) for a devout Muslim to endorse.

## Introduction

There are two different kinds of religious rituals within Islamic scriptural sources. The first kind involves rituals mentioned explicitly in the Qur’an, and almost all Muslim scholars, both Shīʿite and Sunni,^1^ agree about their importance and validity. However, the second kind involves rituals about which Muslim scholars of similar knowledge, standing, or authority have peer disagreements about how to interpret them. In some cases, they may even have completely opposite judgments. I argue in this paper that rituals of the latter kind—that is, the ones that are subject to rational peer disagreement among similarly qualified scholars—can justifiably be seen, even within a purely religious worldview, as non-essential and contingent rituals.^2^ On this basis, a sincerely pious Muslim could be justified in declining to perform or endorse/authorize such a ritual. Moreover, I will argue that rational peer disagreement about the status of certain rituals within a Muslim religious context has important philosophical and practical implications.

Among different rituals that Muslims practice, some rituals such as Ṣalāt (daily prayer), Ṣawm (fasting), Ḥajj (pilgrimage), and Zakāt (alms tax) are generally non-controversial. However, some practices, such as circumcision (Khitān) and veiling (Hijāb), are two of the most controversial Islamic practices among Muslims and non-Muslims alike.^3^ Although they are completely different practices, both circumcision and veiling raise a similar epistemic challenge^4^: could a devout Muslim be religiously justified in declining to perform them, for example, on moral grounds? To be sure, the majority of Muslims believe that the Qur’an endorses ḥijāb for women as a veil that is supposed to cover the head and chest; but there is a trend among Muslim scholars (either jurists or intellectuals)^5^ stating that a head-covering ḥijāb is not *necessarily* religiously obligatory.^6^ These intellectuals refer to certain interpretations of the Qur’an with an argument that ḥijāb essentially aims for modesty and a devoted religious woman can be a modest person even if she is not wearing a ḥijāb.^7^ What about circumcision? Can a similar sort of analysis be applied? Although the majority of Muslims practice at least male circumcision, with a smaller proportion practicing both female and male genital cutting in concert (see below), my question here is whether a well-informed, rational and perfectly devout Muslim could—using the hermeneutical resources of Islam itself—decline to perform or authorize the circumcision of a male child who is too young to consent for himself, as is widely accepted in the case of female children. I will answer in the affirmative.^8^

## Circumcision: What is it and Why is it Contentious?

First, we must clarify what circumcision refers to. In English, the term “circumcision” is most commonly used to refer to male circumcision specifically, that is, the partial or total removal of the penile prepuce or foreskin. However, the Arabic word for circumcision—“khitān”—is gender-neutral and can be used to refer to the cutting of either the male or female prepuce (Box 1). In the Western world, any cutting of female genitalia that is not considered medically necessary^9^ is usually termed “female genital mutilation” or FGM, following the definition of the World Health Organization (WHO, 2008). However, the WHO definition includes some forms of ritual female genital cutting (FGC) that are less physically substantial than the male circumcisions carried out within the same families, raising questions about sex-based categorizations (O’Neill et al., 2020). According to one interpretative tradition, girls within the practicing subsets of Muslims are considered equally “worthy” of being circumcised as are boys (albeit by means of a less intrusive procedure), thus marking a break with the older Jewish covenantal ritual from which girls are, by contrast, excluded--arguably due to having a lower status than males within classical rabbinical Judaism (Shweder, 2021; Cohen, 1997). In the case of Islam, the forms of FGC in question include so-called ritual “nicking, pricking, or partial removal of the clitoral prepuce or hood”—the most common forms of FGC in some “parts of South and Southeast Asia,” where they are carried out, alongside male circumcision, for religious reasons within some sects of Islam (Bootwala, 2019; Dawson et al., 2020; Duivenbode & Padela, 2019; Earp et al., 2021; Earp, 2022b; Rashid et al., 2020).

**Box 1. A brief overview of the human prepuce: male, female, intersex.** Adapted with permission from Myers and Earp (2020) and Earp (2022a, b). The genital prepuce is a shared anatomical feature of both male and female members of all human and non-human primate species (Cold & Taylor, 1999). In humans, the penile and clitoral prepuces are undifferentiated in early fetal development, emerging from an ambisexual genital tubercle that is capable either of penile or clitoral development regardless of genotype (Baskin et al., 2018). Even at birth—and thereafter—the clitoral and penile prepuces may remain effectively indistinguishable in people with certain intersex traits or differences of sex development (Fahmy, 2015; Hodson et al., 2019; Pippi Salle et al., 2007). The prepuce is an integrated feature of the external genitalia, having evolved to function in concert with other genital structures; for example, it forms the anatomical covering of the glans penis or clitoris, thereby internalizing each and “decreasing external irritation and contamination” (Cold & Taylor, 1999, p. 34). In the case of the penile prepuce, an additional function—alongside its biomechanical role in sexual intercourse (Purpura et al., 2018)—is to protect the urinary opening from abrasion, as this runs through the penile, but not the clitoral glans (Fahmy, 2020). The penile prepuce has a mean reported surface area of between 30 and 50 square centimeters in adults (Kigozi et al., 2009; Werker et al., 1998) and is the most sensitive part of the penis, both to light touch stimulation and sensations of warmth (Bossio et al., 2016; Sorrells et al., 2007). The clitoral prepuce, while smaller in absolute terms, is continuous with the sexually sensitive labia minora; it is also an important sensory platform in its own right, and one through which the clitoral glans can be stimulated without direct contact (which can be unpleasant or even painful) (O’Connell et al., 2008). In both sexes, the human prepuce is “a specialized, junctional mucocutaneous tissue which marks the boundary between mucosa and skin [similar to] the eyelids, labia minora, anus and lips … The unique innervation of the prepuce establishes its function as an erogenous tissue” (Cold & Taylor, 1999, p. 34).

In deciding what terminology to employ in this context, one must confront the fact that ritual male genital cutting (MGC), in contrast to ritual FGC, has not been defined as “mutilation” by any Western organization even when considering its most dangerous forms (O’Neill et al., 2020). For example, ritual male circumcision as practiced by the Xhosa of South Africa frequently leads to scarring and perceived disfigurement and carries a high rate of penile amputation and death, often due to sepsis or suicide (Earp et al., 2017; Douglas & Nyembezi, 2015; van der Merwe, 2020).^10^ Why it is that no form of medically unnecessary MGC, but all forms of medically unnecessary FGC, are considered to be “mutilating” by the WHO, irrespective of the actual extent of cutting in either case or any associated physical-functional implications, remains unclear.^11^ However, some scholars argue that such disparate labeling may reflect a Western evaluative bias,^12^ given that MGC, but not FGC, is familiar to Western culture (Darby, 2016; Njambi, 2004; Oba, 2008; Tangwa, 2004). Historically, this would have been due to contact with Judaism (Glick, 2005), but the driving force today seems to be medicalized newborn male circumcision as it is practiced in the United States and to a lesser extent in Canada (Darby, 2013; Earp, 2016; Gollaher, 2000). As Toubia (1999, p. 5) notes:

A major difference between male and female circumcision is that the female procedure is primarily carried out in Africa [among other regions of the Global South], which is currently the least dominant culture in the world. The male procedure is also common in the same countries, but it is also common in the United States, which is currently the most dominant culture in the world through its far-reaching media machine. This historical situation has made it easier to vilify and condemn what is common in Africa and sanctify what is popular in America.^13^

Within a Muslim religious context, both male and female circumcision are controversial in the sense that neither is ordained nor even mentioned explicitly in the Qur’an. However, in addition to the Qur’an, there are other sources of Islamic legislation, such as prophetic Hadiths (i.e., sayings and deeds attributed to the Prophet). The Qur’an itself does not contain all relevant rulings. Many issues in Islamic Sharīʿa law rely not on the Qur’an but on the Hadiths. For example, although the prayers, subject to interpretation, are considered by many as one of the “essential” pillars of Islam, the number of prayers, their parts, and how to pray are not mentioned in the Qur’an. Circumcision is an analogous issue in the sense that the primary source of its interpretation is Hadith.

There are two broad schools of thought regarding male circumcision within Islamic jurisprudence. One holds that it is a religiously obligatory practice (the majority position), and another holds that it is merely preferred or recommended (argued by a few jurists). For instance, relying on Al-Ghazali’s *Iḥyā’ ‘ulūm al-dīn*, in his *al-Maḥajjat al-bayḍāʾ*, Mulla Muhsin al-Fayḍ al-Kāshānī reports various Hadith on male circumcision. Some of them consider circumcision as *wājib* (obligatory), meaning that it is a religious duty commanded by God, so the one who does it will deserve a reward, and the one who fails to do it will be punished in the afterlife. Some of them consider it *mustahabb* (recommended), meaning that it is not essential to be done though its fulfillment is rewarded, and negligence will not be punished (1960, vol. 1, 524–526).

It is also reported from the Prophet in *Ṣaḥīḥ al-Bukhārī* (5891) that male circumcision, like shaving the pubic hair, is among those activities recommended for a Muslim to do for hygiene and purification. Likewise, in answering the question of whether male circumcision is required of parents to do or authorize for their children, Ayatollah Sistani, a prominent Shīa *Marji’ Taqlīd* (religious authority), replies that it is recommended and not obligatory.^14^ In the same line of thought, from an Islamic perspective, one can argue that male circumcision *can* be suspended until the boy (at least) reaches puberty and decides for himself.^15^

By contrast, in Islamic jurisprudence, opinions endorsing female circumcision typically are regarded as being less authoritative than those endorsing male circumcision. One reason for this view is that while male circumcision has been mentioned in many prophetic Hadiths, the Hadiths concerning female circumcision are relatively few in number and can more readily be judged as weak or dubious according to rules established by scholars of the Hadiths.^16^

Another argument for distinguishing male and female circumcision in terms of their respective statuses in Islam, considered globally, is that female circumcision is not a common practice in most Muslim-majority countries, whereas male circumcision is ubiquitous within the same countries. Therefore, it is commonly argued that “female circumcision is not really a ‘religious’ practice” (i.e., inherent to, or necessary within, the religion; otherwise, it would be more widespread) “but is rather ‘merely’ a cultural practice” that has only incidentally become associated with Islam in certain contexts (Earp, 2021).^17^ In support of this view, it is sometimes mentioned that female genital cutting existed in Africa prior to the advent of Islam and was incorporated into Muslim ritual practice in some, rather than all, places where Muslims have large established populations.^18^

A potential problem with this argument is that male genital cutting also existed in Africa prior to the advent of Islam—and indeed Judaism—and was eventually adopted by various Semitic tribes, including the ancestors of both Muslims and Jews (Cohen, 1997). Moreover, there may be alternative explanations^19^ for the near-universality of male, but not female, circumcision within these faith communities that do not amount to a dichotomous distinction between “religious” (male) and “merely cultural” (female) genital cutting in situations where Muslims practice both together. Indeed, the very distinction between “religious” and “cultural” as descriptions of certain practices has been questioned by anthropologists and philosophers. For example, as Brusa and Barilan (2009) argue:

In the context of circumcision, we find the moral dimension of the distinction between the ‘religious’ and the ‘cultural’ rather tenuous. Research on the historical development of circumcision demonstrates very intricate links bridging religion, institutions of social power and metamorphoses in meaning and practice over time and space (p. 471).

Nevertheless, many Muslim scholars (both jurists and intellectuals) believe that female circumcision is not religiously obligatory for devout Muslims. For example, in the same *fatwā* mentioned above, Ayatollah Sistani mentions that female circumcision is *haram* (forbidden) if it is harmful. Whether it is harmful or harmful enough to fall within this proscription is a matter of ongoing debate (see, e.g., Rogers, 2016). Sheikh Muhammad Sayyid Tantawy of Al-Azhar also argues against female circumcision as an un-Islamic ritual because it has nothing to do with religion.^20^

In keeping with this perspective, I will assume that a devout Muslim could be justified in declining to perform or authorize such a procedure for their daughter. In the case of male circumcision, however, such an argument seems harder to make. This is because the majority opinion among Muslim jurists and intellectuals with respect to male circumcision is the very opposite of what I have just said about female circumcision: they regard it as religiously required. I believe an argument can be made against this requirement, however, by appealing to the logic of peer disagreement, as I explain in the following sections.^21^

## Grappling With Peer Disagreement

As noted, the main reason for such disagreement is that the issue of circumcision, whether female or male, is not mentioned explicitly in the Qur’an. Since the issue of circumcision is not grounded in the Qur’an, within the principles and methodology of Islamic jurisprudence (*usul al-fiqh*), Muslim jurists derive their judgments (*ahkam*) from other sources, such as the Sunnah (Hadith) which is what the prophet—and *Imams* in the Shīʿite school—reportedly said, did or agreed to; *ijma*, which is the consensus of Islamic scholars; and ‘*aql*, which is the power of reason or the rational mind (although ‘aql is not a source of Sunni *usul al-fiqh*). The absence of circumcision in the Qur’an makes it at least liable to different interpretations and opinions among scholars.

On one interpretation, based on Shīʿite and Sunni jurisprudential texts, male circumcision before the age of seven is not obligatory in Islam, and it is *merely* preferred or recommended (*mustahabb* or *mandub*), or a kind of custom, according to the *ulemā* (Muslim jurists). In addition, men who convert to Islam *could* be circumcised if they wished; hence there is no requirement to be circumcised.^22^

However, it is agreed among many *fuqahā* (jurists) that male circumcision is obligatory for those who want to become the imam in performing prayer, i.e., the one who leads the congregation in ṣalāt.^23^ It is also agreed that circumcision (mostly for men) is obligatory in the moment of Hajj.^24^ In effect, as one of the Islamic rituals of pilgrimage, Muslims who want to go to Mecca for Hajj (Ṭawāf) are required to be circumcised beforehand. However, since Hajj is not obligatory (*wājib*) for those who cannot afford it, and becoming an *imam* is voluntary, one might argue that circumcision is not always obligatory for everyone.

Sunni and Shīʿite jurists have different opinions on whether male circumcision is obligatory or an act of Sunna—i.e., not obligatory but highly recommended. Among Sunni schools, although Shafiites generally believe that male circumcision is obligatory, Hanbalites, Ḥanīfis and Mālikis believe that male circumcision can be obligatory. In particular, Imam Abū Ḥanīfa and Imam Mālik considered male circumcision as a confirmed Sunnah (*Sunnah Mu’akkadah*)—not obligatory but highly recommended (al-Ḥaṭṭāb 1995; al-Humām 1999; Alahmad & Dekkers, 2012). Some, but not other, Shīʿites regard male circumcision before the age of seven as non-obligatory but preferred or recommended (*mustahabb* or *mandub*).^25^

So understood, it seems plausible to argue that since the necessity of circumcision in Islamic *Sharīʿa* law both for male and female Muslims has been challenged by a number of Shīʿite and Sunni *ulemā*, it may be the case that being circumcised, or authorizing circumcision for one’s children, is not a necessary part of being a Muslim. In other words, one can be a devout Muslim—in good standing, so to speak—and yet not practice the circumcision ritual simultaneously. But why, exactly, does disagreement among *ulemā* give us a philosophical reason to conclude that circumcision can be unnecessary?

## Peer Disagreement Regarding Harm Being Divinely Ordained^26^

What is the difference between rituals that are mentioned explicitly in the Qur’an and practices that are not mentioned? Is there any philosophical or theological significance here? One of the philosophical consequences of this difference has to do with recent discussion in social epistemology concerning “peer disagreement” (*ikhtilāf*).^27^ Within Islam, rational peer disagreements are especially likely to arise when we have rituals and practices that are not mentioned explicitly in the Qur’an.^28^ The peer disagreements will be especially intense if the notion of harm is involved. For example, in the debate on circumcision, there is a possibility that causing harm to a child is, in fact, permissible if divine ordination can be ascertained, while others would argue that a practice cannot be imposed on children if it is harmful on balance or overall.^29^ Let us first explain what peer disagreement is.

Suppose person A believes p while person B believes not-p. Suppose further that they are referring to one thing (religious texts) as evidence supporting their respective claims and that they have equal reasoning skills. Since the two propositions believed are contradictory, one of A or B must be wrong (at least on classical models of logic relying on the law of non-contradiction). However, suppose that both A and B are rational based on their *prima facie* justification, and since they have equal reasoning skills (by stipulation), they should respect one another as reasoners. What should be done in the face of such rational peer disagreement?

Given that both A and B have the same primary (textual) evidence, one possibility is that they should proceed to revise their initial beliefs in light of other sources, e.g., ethics, philosophy, science, etc. A’s and B’s initial views about the proposition believed (p) have equal weight, suggesting that, upon learning of the other’s contradictory belief, they should become less confident in their own opinions. However, both A and B are rationally required to change their views in light of further moral, philosophical and scientific assessment. In this, they could perhaps pursue a process of *reflective equilibrium* as a method (described below). If there are still disagreements after further assessments, both parties then have “equal weight” views. It is important to note that not *any* form of disagreement requires us to rationally change our beliefs.

The reflective equilibrium method seeks to achieve coherence, normatively, between our considered beliefs and judgments about particular cases and the general theoretical principles or rules that explain or justify such judgments. We aim to revise our particular beliefs and judgments, and general principles through reflective equilibrium to reach a plausible and rational coherence among them. As Gaut describes,

reflective equilibrium is not a theory, but a method, of justification; it tells one how to justify one’s moral beliefs, by attempting to render consistent one’s moral principles with one’s judgments about particular cases (2002, 139–140).

The process of reflective equilibrium is not only for modifying our prior beliefs. Rather, we can also add new normative beliefs to our theoretical framework. In practical contexts, reflective equilibrium could help us reach a conclusion about what we *should* do when we are unsure what to do (Scanlon, 2002).

A and B could seek reflective equilibrium (roughly, coherence) between their beliefs at different levels of generality. In effect, their very general beliefs need to cohere with their less-general beliefs and so on, down to their particular judgements. They can get their justification from reflective equilibrium even if they do not have enough evidence that they are correct. This entails that the pursuit of reflective equilibrium sometimes reveals an intuition or judgment that we had not previously had. For example, person A might have had a belief about the necessity of performing circumcision in every situation, but then is brought to see that there may not be such a strict duty, insofar as he maintains his firm moral judgments about broader principles regarding unnecessary harming, or in reasoning by analogy to a wider range of cases, and wishes to have those broader principles make sense. For another example, B might not initially have seen a moral duty not to inflict unnecessary harm. Still, she comes to see there must be such a duty for her firm moral intuitions about various particular cases to be correct. And this, in turn, might count against the belief that circumcision is *always* a duty even if it is divinely ordained.

Both A and B are considering the issue of divine command and the possibility of harm being divinely ordained. They are dealing with an example of inflicting harm (in the sense of a bodily injury, accompanied by pain, effectuated through cutting as in circumcision) and asking whether God’s command-- if that is what it is-- can be justified. However, they might ask themselves what should be done when God tells us to cause harm, and His command does not seem morally to make sense (e.g., to injure and cause pain to a sensitive part of the body when it is not medically necessary to do so). Although they might have different strategies to check and balance their religious beliefs or interpretations with their moral judgments, in any case, they need to seek reflective equilibrium. For instance, suppose that A believes in the general principle of *always* obeying the commands of Sharīʿa laws because he thinks that God completely endorses these Sharīʿa laws and that obeying the commands of Sharīʿa laws is morally good. However, B argues that obeying divine command is not *always* morally obligatory, particularly if we have a strong moral reason, *ceteris paribus*, to support the idea that a practice inflicts unnecessary or unjustifiable harm and hence is morally problematic. These views may come into conflict. If they do, A and B should seek reflective equilibrium between their beliefs. For example, A will have several choices. He can discard his particular belief for one that can be better justified rationally (for example, discard a particular Sharīʿa law if there are strong moral reasons against it), or modify his general principle (for example, only obeying the Qur’an or choosing a different interpretation of circumcision). Some Shīʿite scholars believe that some judgments such as jihād and Friday ṣalāt can be suspended until Imam Mahdi, the prophesied redeemer of Islam who is in occultation, arrives.^30^ Person A might conclude in favor of suspending circumcision following this line of argument. From B’s perspective, he might want to reconsider his excessive rationalism, if at all. In cases where A’s and B’s beliefs are mutually exclusive, then both should reduce their confidence in their respective beliefs and/or seek alternative interpretations. 

## Sociocultural Understanding of Harm

Let us now turn to the question of 'harm'. One might wonder how should harm be understood in this context when harm is, allegedly, being divinely ordained? What are the implications for male or female circumcision? There are many Prophetic sayings about which scholars have disagreements concerning etiology and reliability. For example, Abū Ḥanīfa, the eponymous founder of the Sunni Ḥanafi School, believed that there are very few Hadiths that we can historically trust (Syamsuddin, 2001). One of these Hadiths, which almost all Islamic scholars refer to and on which many *Sharīʿa* laws are based, is the “principle of harm”: let there be no harm nor reciprocating harm (*lā darar wa lā dirār*).

Subjectively speaking, most circumcised Muslims, whether male or female, do not seem to regard themselves as net harmed or harmed on balance by virtue of their circumcision. Some may not regard themselves as harmed, even in a purely physical sense. Others may concede that, to some extent, their body has been harmed while still believing that psychosocial or religious benefits outweigh those harms so that they are not harmed in the aggregate or overall. For example, in Islamic *usul al-fiqh*, it is generally argued that cutting any part of the body in itself is, *prima facie*, forbidden because it is harmful. However, it would be permissible if there was a utility (*maslaha*) involved in doing that. So, cutting any part of the body is *only* permissible if its utility is more than its harm. To understand this phenomenon, one needs to have an account of harm and to understand the sociocultural and epistemic context in which circumcision occurs.^31^

According to philosopher Scott Campbell (2006, p. 226–227),

one of the components of risk is harm. This is the level of badness or loss associated with the occurrence of x. Harm does not just include physical injury but any sort of circumstance that P would prefer not to be the case. If there is nothing bad about x at all then P is not at risk from x. This entails that our attitudes and preferences partly determine risk, because our attitudes and preferences determine what counts as a harm.

Here P refers to a person and x to an action or occurrence. In Campbell’s view, once we understand our subjective attitudes and preferences, we will be able to highlight the objective nature of certain harms. As a consequence of this, Campbell argues, in many cases, it will not be possible to know for sure whether someone is at risk of harm or has actually been harmed until we know their relevant (considered) attitudes and preferences. With respect to circumcision, one’s attitudes and preferences will likely be informed by what is culturally normative in their environment; but attitudes and preferences vary both within and between cultures, and they are subject to changing over time: for example, as acquires new information or learns a new perspective. If one grows up in a culture where the prepuce, whether male or female, is widely believed to be a “useless flap of skin,” one may intuitively assign a value of zero to this tissue, such that its being damaged or removed will not seem a harm. But as people are “exposed to and learn about different cultural assumptions and practices regarding cut versus uncut genitalia—whether through travel, reading, or surfing the Internet—they may come to regard the majority practice of their own group as being harmful or otherwise problematic, and consequently re-assess the value of their own genital status” (Earp & Darby, 2017, p. 25; Barutcu, 2022).

For example, a man might discover that the penile foreskin, rather than being a “useless flap of skin,” may be “the most sensitive part of the penis to light-touch sensation” (Bossio et al., 2016; Earp, 2022a, b; Sorrells et al., 2007), or that its manipulation in sexual contexts affords particular subjective sensations that many men with foreskins report finding valuable (Ball, 2006). If such a man reached the conclusion that, in fact, he had been harmed by virtue of having his foreskin removed, this would not be obviously unreasonable.^32^ Analogously, consider this account from a woman in the Dawoodi Bohra community who, in adulthood, came to reflect on her childhood circumcision and see it in a new light (quoted in Taher, 2017):

… I feel robbed and cheated of my sexuality, and feelings of inadequacy and incompleteness remain with me till today, even at the age of 61.

Insofar as it is reasonable for a Muslim person, whether male or female, to regard themselves as having been harmed by virtue of having had their genital prepuce cut for ritualistic reasons as a child, the “principle of harm” Hadith might seem to weigh against the practice of non-voluntary and non-therapeutic circumcision. However, suppose someone, for example, happens to believe that the potential benefits of male circumcision, such as reducing the transmission of venereal diseases, including AIDS (as recommended by WHO for adults, voluntary circumcision), outweigh the harm involved in performing circumcision. In that case, he is entitled to act from his justified reason as long as he is adequately informed and *autonomously* decides to do that.

## Conclusion

Peer disagreements are likely to arise when we have rituals that are *not* mentioned explicitly in the Qur’an. In this essay, I have argued that (1) rituals (not explicitly prescribed in the Qur’an) which are subject to rational peer agreements among Muslim jurists/scholars can plausibly be seen as non-obligatory, non-essential and contingent, (2) male circumcision is not explicitly prescribed in the Qur’an, and it is subject to rational peer agreement, (3) therefore, male circumcision is non-obligatory, non-essential and contingent. I have shown in this essay that rational peer disagreement about the status of certain rituals within a Muslim religious context has important philosophical and practical implications. Those religious rituals subject to rational peer disagreement among similarly qualified scholars can plausibly be seen as non-obligatory, non-essential and contingent rituals to practice. The mere fact of having a rational peer disagreement can make us, at least, suspend extra-Qur’anic rituals. Thus, a religious Muslim could be rationally justified in declining to perform such rituals. The existence of rational peer disagreement across scholars can provide a good reason to doubt the necessity of practicing such rituals. However, this does not entail that any form of disagreement provides an acceptable reason to decline to act.

Following my argument in this paper, we can conclude that religious practice in connection with circumcision (mostly male) among the larger Muslim community is not authoritative since the Qur’an does not prescribe it as a necessary practice. This makes it plausible to maintain philosophically (or rationally) that circumcision is not obligatory in one’s claim to be a member of the Muslim community. Additionally, based on differences of opinion among Muslim jurists in regard to interpreting the textual sources like the Sunna, in the context of juridical methodology that applies the principle of avoiding harm and promoting the public good, the practice can be regarded as unnecessary or simply recommended (mandub, mustahabb) like a number of cultural practices in Muslim societies.

So understood, as far as circumcision is considered, my argument implies that it is not morally and religiously *necessary* to perform circumcision in Islam. The reason is since circumcision is not mentioned explicitly in the Qur’an, and there are rational peer disagreements among Muslim scholars about whether circumcision must be performed or not. Sunni and Shīʿite jurists and intellectuals have contradictory opinions on whether male or female circumcision are obligatory or acts of Sunna. So, it seems plausible to conclude that the necessity of circumcision in Islamic *Sharīʿa* law both for male and female Muslims has been challenged by a number of Shīʿite and Sunni *ulemā*, and that circumcision is not a *necessary* part of being a Muslim.

## Footnotes

1 For more details on these two main branches of Islam and their philosophical and jurisprudential differences see: Gleave (2018) and Hallaq (1997, chs. 2–3).

2 For more on the distinction between the essential and the accidental or contingent in Islam, see: Soroush (2009, Ch. 4). Note that all rituals irrespective of their reference in the Qur’an are subject to rational peer disagreement among Muslims. Historically speaking, for example, not all Muslim communities have considered contingent what other Muslim communities may have considered essential. However, in this essay, I will focus on those rituals that are not explicitly mentioned in the Qur’an.

3 All rituals may be considered as practices, but not all practices may be considered as rituals. Circumcision may be considered as a ritual and a practice, but veiling is a practice rather than a ritual.

4 There is also a difference between enforcing “hijab” (which is textually anchored in the Qur’an) and children’s circumcision, the latter is known to cause psychological damage to the future of children’s well-being.

5 Muslim intellectuals generally are not jurists. Both jurists (*ulemā*) and non-jurists are scholars though.

6 See Mernissi (1992, Ch. 5). Shahrur (2009, Introduction and Ch. 5).

7 Ahmad Qabil, as a prominent Shīa scholar, thinks so. See his e-book *Shari’at-i ‘aqlani* [Rational Religion] on his website: http://www.ghabel.net/shariat/. See also Ridgeon (2020) and Rezaei (2020).

8 My argument in this paper is philosophical. It does not necessarily answer psychological and sociological questions such as to why Muslims still practice circumcision if it is not religiously necessary.

9 See e.g., BCBI (2019) as a recent international consensus statement.

10 These and other examples of relatively severe forms of male genital cutting are summarized in Shahvisi and Earp (2019). For further inconsistencies in the WHO policies on child genital cutting practices, see Earp and Johnsdotter, (2020).

11 I will refer to both sets of practices as “genital cutting” or as “circumcision” where appropriate, preferring to leave the decision about whether one’s own altered genitalia should be described as mutilated to each affected individual (see Earp, 2019).

12 See Johnson (2010).

13 Another potential difference is that MGC, but not FGC, has become culturally associated with health benefits in Western contexts, including a reduction in the risk of acquiring certain infections (although the existence, likelihood, and/or magnitude of these benefits is controversial in the medical literature). For an alternative view, see Earp (2021).

14 See his website: https://www.sistani.org/persian/qa/0896/

15 See e.g., Dabbagh (2017).

16 Gomaa (2013). I would like to thank the anonymous reviewers for raising this critical point.

17 See e.g., Bewley and Momoh (2010). Also see: Odukogbe et al. (2017).

18 Thanks to the editor for raising this point.

19 In brief, one explanation holds that males have had much greater social, political, and legal status than females throughout most of the respective histories of Judaism and Islam. Since circumcision usually is considered a status-elevating rite within practicing groups (e.g., a sign of a divine covenant within Judaism), women and girls have more typically been left out. With respect to Jewish practice, for example, as Cohen (1997) notes: “Of all the rituals from which women are excluded by rabbinic culture, the exclusion from circumcision is at once the most obvious and the most problematic” (p. 560). This suggests that it is the exclusion of females from ritual circumcision that “reflects their lower status” in certain contexts (Earp, 2022b). Nevertheless, in the subset of Muslim communities where they are, in fact, included in the institution of circumcision alongside males, it does not follow that the practice ceases to be “religious” in nature only in the case of females.

20 See http://news.bbc.co.uk/1/hi/42914.stm. See also Kassamali (2005).

21 See e.g., Najafi (1981, Vol. 31, pp. 260–263 and 257) and Alahmad and Dekkers (2012).

22 See e.g., Khomeini (1987, Vol. 2, p. 310). See also: Najafi (1981, Vol. 31, pp. 262–3) and Ameli (1994, Vol. 24, p. 271).

23 See e.g., Najafi (1981, Vol. 13, pp. 384–385).

24 See e.g., Najafi (1981, Vol. 19, p. 274). However, there are some alternative views on whether circumcision is required for Hajj. See, e.g., Ameli (1991, Vol. 8, p. 118).

25 See e.g., Najafi (1981, Vol. 41, 672) and Tusi (1973, Vol. 8, 67).

26 Parts of my argument in this section and the next section have been discussed before in an interview with Helen De Cruz in the Prosblogion, a philosophy of religion blog. The blog, however, is not available anymore.

27 Christensen (2007) and Christensen (2013).

28 There are also some peer disagreements about rituals and practices that are mentioned in the Qur’an. For instance, as noted above, although ḥijāb is mentioned in the Qur’an, there are different views as to whether covering the head is necessary.

29 I would like to thank the anonymous reviewers for raising this critical point.

30 For a similar discussion, see: Alishahi Tabriz, Dabbagh & Koenig (2016). See also Ghassemi (2009) and Akbar (2018).

31 For more on this, see: Sachedina (2009, Introduction).

32 Concerning the potential damage that can be caused in male circumcision, please see this recent personal story about a botched circumcision in the *New Yorker*: https://www.newyorker.com/magazine/2021/10/11/a-botched-circumcision-and-its-aftermath.
